# Acute rheumatic fever in the UK and Ireland: a BPSU surveillance study

**DOI:** 10.1136/archdischild-2024-328277

**Published:** 2025-05-16

**Authors:** Māia Gourlay-Gudex, Mary Salama, Rosie Crane, Elizabeth Whittaker, Tom Parks

**Affiliations:** 1 Imperial College Healthcare NHS Trust; 2 Birmingham Women’s and Children’s NHS Foundation Trust; 3 City St George’s University of London; 4 Imperial College London

Higher rates of childhood *Streptococcus pyogenes* (GAS) infections following the COVID-19 pandemic have exposed uncertainty about the epidemiology of immune-mediated complications of GAS in high-income settings.^1^ Acute rheumatic fever (ARF) is of primary concern due to its potential to cause life-threatening carditis. Early recognition of ARF is critical to prevent progression to rheumatic heart disease, a significant cause of premature death globally.

Accordingly, we reanalysed data from a hitherto unpublished British Paediatric Surveillance Unit (BPSU) ARF study run from May 2015 to May 2016. Briefly, UK and Republic of Ireland (RoI) consultant paediatricians were asked to provide monthly reports and details of: “Any cases of children or young people 0-16 years of age with either a confirmed or suspected new diagnosis of acute rheumatic fever seen in the past month.” Subsequently, we categorised reported cases into: ‘confirmed ARF’ using the Jones Criteria for ‘low-risk’ populations^2^ (Box 1); or ‘possible ARF’ based on either sufficient clinical criteria without evidence of preceding GAS, or children with evidence of preceding GAS with carditis or polyarthritis but only one minor criterion.

Over 13 months, 38 cases were reported to the BPSU (all from the UK): 28 were categorised as confirmed ARF and six as possible ARF (none recurrent). Four did not meet diagnostic criteria (one due to missing data) and were removed from further analyses. Using a combined UK and RoI mid-study denominator estimate of 14,209,901 children aged 0-16 years (online supplemental appendix 1), we estimated a confirmed ARF incidence rate of 0.18 per 100,000 person-years (online supplemental appendix 2). Among the 27 confirmed cases with demographic information available: 14 were female; the median age was 10.2 years (interquartile range 6.8-12.3), the youngest a child with chorea at age 2.9 years diagnosed by a paediatric neurologist; 26 had been born in the UK (one not recorded); 20 reported White British or White Irish ethnicity, the remainder from Mixed, Asian or Unknown ethnicities. Preceding GAS infection was evidenced by elevated antistreptolysin-O titres in 25 confirmed ARF cases (GAS also cultured from a throat swab in eight) while the remaining three children had chorea without evidence of preceding GAS infection. Carditis demonstrated on echocardiography was observed in 18 of the confirmed ARF cases including 10 with moderate to severe mitral and/or aortic regurgitation (online supplementary appendix 3). Additionally, two children with possible ARF had carditis both with severe regurgitative lesions (Box 2). There were 18 cases of chorea (incidence 0.12 per 100,000 person-years) of whom six had no additional major criteria.

In this most recent UK surveillance, ARF remained a rare disease, consistent with previous studies from high-income settings. For context, our incidence estimate is 100-fold less than prospective ARF surveillance in low income settings^3^ and 10-fold less than that of Kawasaki disease in the UK, an analogous inflammatory disease with cardiovascular complications. Nonetheless, the predominance of severe carditis and chorea, with similar incidence to a more recent BPSU survey,^4^ raises the possibility of under-diagnosis while underscoring the difficulty in recognising early or subtle features of ARF.^1^ Ultimately, pending further surveillance, clinicians must remain vigilant as a small number of children will continue to present with ARF in the UK.

## Supplementary Material

Supplementary Appendix

## Box 1 American Heart Association Revised Jones Criteria for Acute Rheumatic Fever (ARF) in low-risk populations

Initial (non-recurrent) ARF is diagnosed in low-risk populations^A^ where a child has evidence of preceding Grp A Streptococccal infection^B^ PLUS two major OR one major and two minor criteria.

Major criteria:

- Carditis^C, D^
- Polyarthritis
- Chorea^D^
- Erythema marginatum
- Subcutaneous nodules

Minor Criteria:

- Polyarthralgia
- Fever ≥38.5°C
- Erythrocyte Sedimentation Rate >60mm or C Reactive Protein >30mg/L
- Prolonged PR interval for age

A: Low-risk populations are defined as ARF incidence ≤2 per 100 000 school-aged children or all-age rheumatic heart disease prevalence of ≤1 per 1000 population per year.

B: Elevated or rising antistreptolysin-O or anti-DNAse B titre.

C: If carditis is used as the major criteria, prolonged PR interval cannot be used as the minor criteria.

D: Where chorea or indolent carditis are the major criteria, evidence of preceding GAS is not needed.

(Adapted from reference 2)

## Box 2 Vignettes for two ‘possible ARF’ cases with carditis

11-year-old male without laboratory evidence of GAS infection with two major criteria of carditis (severe mitral and aortic regurgitation) and polyarthritis, plus one minor criterion of elevated CRP (62mg/L).

13-year-old male with preceding GAS evidence from a throat culture with one major criteria of carditis (severe mitral regurgitation and moderate aortic stenosis), and one minor criterion of fever.
