# Supplementary material for: Acute rheumatic fever in the UK and Ireland: a BPSU surveillance study
Source: Arch Dis Child. Author manuscript; Available in PMC 2025 Jan 26. (PMC7617347; doi:10.1136/archdischild-2024-328277)
Supplement: Supplementary Appendix [file EMS202800-supplement-Supplementary_Appendix.pdf]

Online Supplementary Appendices to

Acute rheumatic fever in the UK and Ireland: a BPSU surveillance study

Māia Gourlay-Gudex, Mary Salama, Rosie Crane, Elizabeth Whitaker, Tom Parks

Appendix 1: Denominator population estimates

For our denominator in our incidence calculations, we used estimates of the population of children living in the UK and Republic of Ireland (RoI) made by the Institute for Health Metrics and Evaluation (IHME) [1] averaged over the years 2015-2016 to give a mid-study estimate (Supplementary Table 1).

Supplementary Table 1. **Denominator population estimates from IHME**

Age in years (inclusive)	Country	Year		Study midpoint
		2015	2016	
0-16	RoI	1154900	1155102	1155001
	UK	13013933	13095868	13054900
	Total	14168833	14250969	14209901
5-14	RoI	680947	690419	685683
	UK	7531487	7667825	7599656
	Total	8212434	8358244	8285339

RoI, Republic of Ireland

References

1. GBD 2021 Demographics Collaborators. Global age-sex-specific mortality, life expectancy, and population estimates in 204 countries and territories and 811 subnational locations, 1950–2021, and the impact of the COVID-19 pandemic: a comprehensive demographic analysis for the Global Burden of Disease Study 2021. Lancet. 2024;403:1989-2056. DOI: 10.1016/S0140-6736(24)00476-8

Appendix 2: Incidence calculations

For our primary estimate of ARF incidence, we used the number of cases of confirmed ARF in our study (n=28) annualised to a 12 month period as the numerator divided by an estimate of the combined mid-study UK and RoI children aged 0-16 years as the denominator (detailed in online supplemental appendix 1). We also calculated incidence with the numerator as confirmed and possible ARF combined (n=34), confirmed ARF with carditis (n=18) and confirmed ARF with chorea (n=18). Finally, for comparison with previous studies[1,2], we calculated incidence rates limited to the population aged 5-14 years inclusive (Supplementary Table 2).

Supplementary Table 2. **Incidence of ARF in the UK and RoI during the study**

Case definition	Age range	Reported cases	Annualised cases	UK and RoI Denominator	Incidence rate per 100,000 person-years
Confirmed ARF*	0-16	28	25.85	14209901	0.18
	5-14	24	22.15	8285339	0.27
Confirmed or possible ARF	0-16	34	31.38	14209901	0.22
	5-14	27	24.92	8285339	0.30
Confirmed ARF with carditis	0-16	18	16.62	14209901	0.12
	5-14	17	15.69	8285339	0.19
Confirmed ARF with chorea	0-16	18	16.62	14209901	0.12
	5-14	16	14.77	8285339	0.18

References

1. Okello E, Ndagire E, Muhamed B, et al. Incidence of acute rheumatic fever in northern and western Uganda: a prospective, population-based study. Lancet Glob Health. 2021;9:e1423-e1430.
2. Jackson SJ, Steer AC, Campbell H. Systematic Review: Estimation of global burden of non-suppurative sequelae of upper respiratory tract infection: rheumatic fever and post-streptococcal glomerulonephritis. Trop Med Int Health. 2010;16:2-11.

Appendix 3: Supplementary description of carditis cases

Carditis demonstrated by echocardiography was observed in a total of 18 of the 28 confirmed ARF cases including 10 children with moderate to severe regurgitation of the mitral and/or aortic valve (Supplementary Table 3). Additionally, both of the children with possible ARF with carditis had severe regurgitative lesions potentially compatible with a more chronic rheumatic process. Thus, although not considered in our incidence rate calculations, it is noteworthy that at least one of these children with possible ARF with carditis could have met the Jones Criteria for confirmed ARF under the bracket of 'indolent carditis' where an exception can be made to the requirement for evidence of preceding GAS infection.[1]

Supplementary Table 3. **Severity of carditis cases in children with confirmed or possible ARF**

Valve lesion	Severity	Confirmed ARF	Possible ARF	Total
Isolated MR	Mild	8	0	8
	Moderate to severe	3	1	4
MR and AR	Mild	0	0	0
	Moderate to severe	6	1	7
Isolated AR	Mild	0	0	0
	Moderate to severe	1	0	1
Total	-	18	2	20

MR, mitral regurgitation; AR, aortic regurgitation

References

1. Gewitz MH, Baltimore RS, Tani LY et al. Revision of the Jones Criteria for the diagnosis of acute rheumatic fever in the era of Doppler echocardiography: a scientific statement from the American Heart Association. Circulation. 2015;131:1806-18.
